# Investigating the Relationship Between Metatarsal Primus Elevatus and Hallux Rigidus Using Weight-Bearing Computed Tomography: A Retrospective Comparison Study

**DOI:** 10.7759/cureus.91139

**Published:** 2025-08-27

**Authors:** Hirotaka Fukushima, Makoto Kubota, Tadashi Kimura, Takumi Kihara, Mitsuru Saito

**Affiliations:** 1 Department of Orthopaedic Surgery, The Jikei University School of Medicine, Tokyo, JPN

**Keywords:** hallux rigidus, joint congruency, metatarsus primus elevatus, weightbearing computed tomography, windlass mechanism

## Abstract

Background: Hallux rigidus is a degenerative disease of the first metatarsophalangeal (MTP) joint, characterized by joint space narrowing and dorsal osteophyte formation. Metatarsus primus elevatus (MPE) is frequently observed in hallux rigidus, although its role in the pathogenesis remains unclear. The windlass mechanism, which stabilizes the medial longitudinal arch by tightening the plantar fascia during hallux dorsiflexion, plays a key role in normal foot biomechanics. Dysfunction of this mechanism may result in loss of arch stability and altered loading of the first MTP joint, potentially contributing to the development or progression of hallux rigidus.

Methods: Eighteen patients (21 feet) with Grade I or II hallux rigidus and 10 healthy volunteers (19 feet) underwent weight-bearing CT. Sagittal images were used to measure the width of the MTP joint space and the deviation of the center of rotation. Image acquisition was performed with the hallux in a minimally dorsiflexed position under static weight-bearing conditions to provide standardized and reproducible comparisons. Comparisons between the groups were performed using t-tests and one-way analysis of variance (ANOVA).

Results: The dorsal joint space was significantly narrower in the hallux rigidus group than in the control group (1.32 ± 0.15 mm vs. 1.88 ± 0.11 mm; P < 0.01). The center of rotation was located in a more plantar position in the hallux rigidus group (-0.39 ± 0.17 mm) compared with the control group (0.51 ± 0.09 mm; P < 0.01). These differences were more pronounced with increasing disease severity, suggesting the early presence of MPE.

Conclusions: MPE, as defined by a plantar shift in the center of rotation under load, is present from the early stages of hallux rigidus and may disrupt joint congruity and promote cartilage degeneration. These findings support a central role for MPE and failure of the windlass mechanism in the pathogenesis of hallux rigidus.

## Introduction

Hallux rigidus is a form of osteoarthritis of the first metatarsophalangeal (MTP) joint characterized by joint space narrowing and dorsal osteophyte formation. In this condition, dorsal protrusion of the first metatarsal head under load, referred to as metatarsus primus elevatus (MPE), is commonly observed. Kihara et al. previously reported the potential involvement of the windlass mechanism in such changes [1,2]. The purpose of this study is to use weight-bearing CT to measure joint space narrowing and changes in the positional relationship between the proximal phalanx and metatarsal head in relatively early-stage hallux rigidus, and to further clarify the contribution of MPE and dysfunction of the windlass mechanism to the pathogenesis of hallux rigidus.

## Materials and methods

Participants

This study included 18 patients (21 feet) who presented to our hospital with complaints of pain in the hallux and were diagnosed with hallux rigidus (HR group) based on physical examination by an orthopedic specialist and findings from radiographs and CT scans. Patients were included in the HR group if they met the following diagnostic criteria: (1) tenderness and pain on the dorsal aspect of the first metatarsal head during dorsiflexion, (2) joint space narrowing and osteophyte formation confirmed by imaging, and (3) absence of advanced hallux valgus. All patients were 20 years of age or older, and written informed consent was obtained voluntarily. Patients with a history of rheumatoid arthritis, gout, foot trauma, or other foot-related conditions were excluded. None of the HR patients had received prior intra-articular corticosteroid injections. The control group consisted of 10 healthy adults (19 feet), aged 20 years or older, with no history of rheumatoid arthritis, gout, trauma, or other relevant foot conditions. The same exclusion criteria were applied to the control group. Disease staging in the HR group was determined using the Hattrup and Johnson classification system [3]. This study was conducted in accordance with the tenets of the Declaration of Helsinki and approved by the Institutional Review Board of our institution. Written informed consent was obtained from all participants.

Imaging

A CT scanner capable of simulating weight-bearing conditions was used for the analysis. CT imaging was performed with the toes and the plantar surface of the foot in contact with a flat CT platform, and a load equivalent to body weight was applied to reproduce standing alignment. This weight-bearing CT methodology has been validated in international journals, demonstrating its reliability for foot pathology assessment [1,2,4]. The scans were acquired using an Optima CT660 Pro 64-row CT system (GE Healthcare, Tokyo, Japan) with a slice thickness of 0.625 mm. Other imaging parameters followed standard high-resolution acquisition protocols used in musculoskeletal CT imaging, including a tube voltage of 120 kV, a tube current of 80 mAs, and a matrix size of 512 × 512, optimized for bone detail.

Image analysis

To ensure consistent slice selection, both sagittal and coronal CT images were reviewed to identify the approximate center of the first metatarsal head. The sagittal slice corresponding to this central region, in which the metatarsal head appeared largest and the articular surface, particularly the plantar side, was clearly visualized, was used for measurement.

MTP Joint Space Width

**Figure 1:**
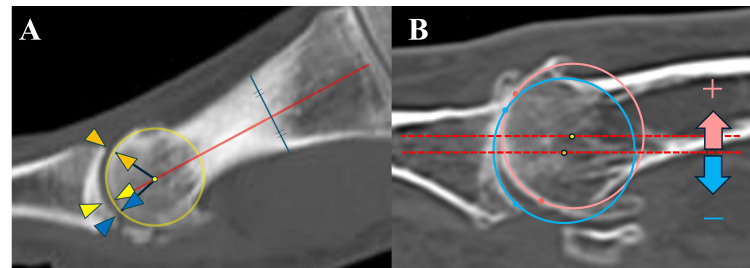
Measurement parameters on weight-bearing CT for hallux rigidus evaluation. (A) MTP joint space width: Measured at three points: the dorsal edge of the proximal phalanx articular surface, the extension line of the first metatarsal shaft axis, and the plantar edge. (B) Deviation of the center of rotation: A best-fit circle was drawn along the articular surfaces of the proximal phalanx and metatarsal head to determine each center of rotation. A negative value was assigned when the center of the proximal phalanx was plantar to that of the metatarsal head.

The width of the joint space was measured at three points: the dorsal and plantar edges of the proximal phalanx joint surface and along the extended axis of the first metatarsal. Areas identified as osteophytes were excluded. The bone axis was defined as a straight line passing through the center of the diaphysis and extending to the center of the metatarsal head, positioned equidistant from the dorsal and plantar cortical margins, and aligned with the long axis of the first metatarsal (Figure 1A).

Center of Rotation Deviation

Two points were plotted on each articular surface of the proximal phalanx and metatarsal head, and a perfect circle was drawn through these points to determine the center of rotation for each bone. Osteophytes and areas with changes in curvature on the plantar side of the metatarsal head were excluded. The distance from each center of rotation to the plantar surface was measured, and the difference between them was calculated. A positive value was assigned when the proximal phalanx center was located above the metatarsal head, and a negative value when it was below (Figure 1B). Although the first MTP joint may exhibit multiple centers of rotation depending on the degree of dorsiflexion [5], our analysis was based on static weight-bearing images and aimed to define a representative center under these conditions.

All measurements were performed by the same orthopedic surgeon to eliminate inter-observer variability. These two parameters were compared between the control and HR groups, and further comparative analyses were conducted among the control, Grade I, and Grade II subgroups. Although intra-observer variability was not formally assessed, anatomical landmarks were identified using multi-planar CT slices, and all measurements were performed with standardized techniques to ensure consistency.

Statistical analysis

This study employed a hypothesis-testing framework. Sample size was calculated using G*Power (Ver. 3.0.10; Heinrich-Heine-Universität Düsseldorf, Düsseldorf, Germany). Based on an a priori analysis referring to the previous study by Gelber et al. [6] with α = 0.05 and 1-β = 0.95, the minimum required sample size was determined to be 22. However, due to limited patient availability during the study period, 21 feet were ultimately included in the HR group. Given the significant findings observed, we consider that the impact of this slight shortfall on statistical power is minimal. Means and standard errors were calculated for the MTP joint space width and the center of rotation deviation. For comparisons between the control and HR groups (Grade I + Grade II), Student's t-test was used for all variables. For comparisons among the control, Grade I, and Grade II groups, one-way analysis of variance (ANOVA) was performed for all variables. When a significant difference was observed, the Tukey-Kramer multiple comparison test was performed. The normality of residuals was assessed using the Shapiro-Wilk test, and homoscedasticity was assessed using plots of predicted values versus residuals. If the normality of the residuals could not be confirmed, the data were logarithmically transformed, and intergroup comparisons were performed after confirming the normality of the transformed residuals. P-values of <0.05 were considered statistically significant.

## Results

Patient background

The HR group consisted of 10 men and eight women, while the control group consisted of six men and four women. The mean age in the HR group was 67.1 ± 9.1 years, and the mean BMI was 23.4 ± 2.5 kg/m^2^. In the control group, the mean age was 61.9 ± 7.2 years, and the mean BMI was 22.5 ± 2.2 kg/m^2^. No significant differences in these parameters were observed between the groups (age: P = 0.054; BMI: P = 0.238). Based on the Hattrup and Johnson plain radiographic classification, 12 of the 21 feet in the HR group were classified as Grade I, and nine were classified as Grade II.

MTP joint space width

**Figure 2:**
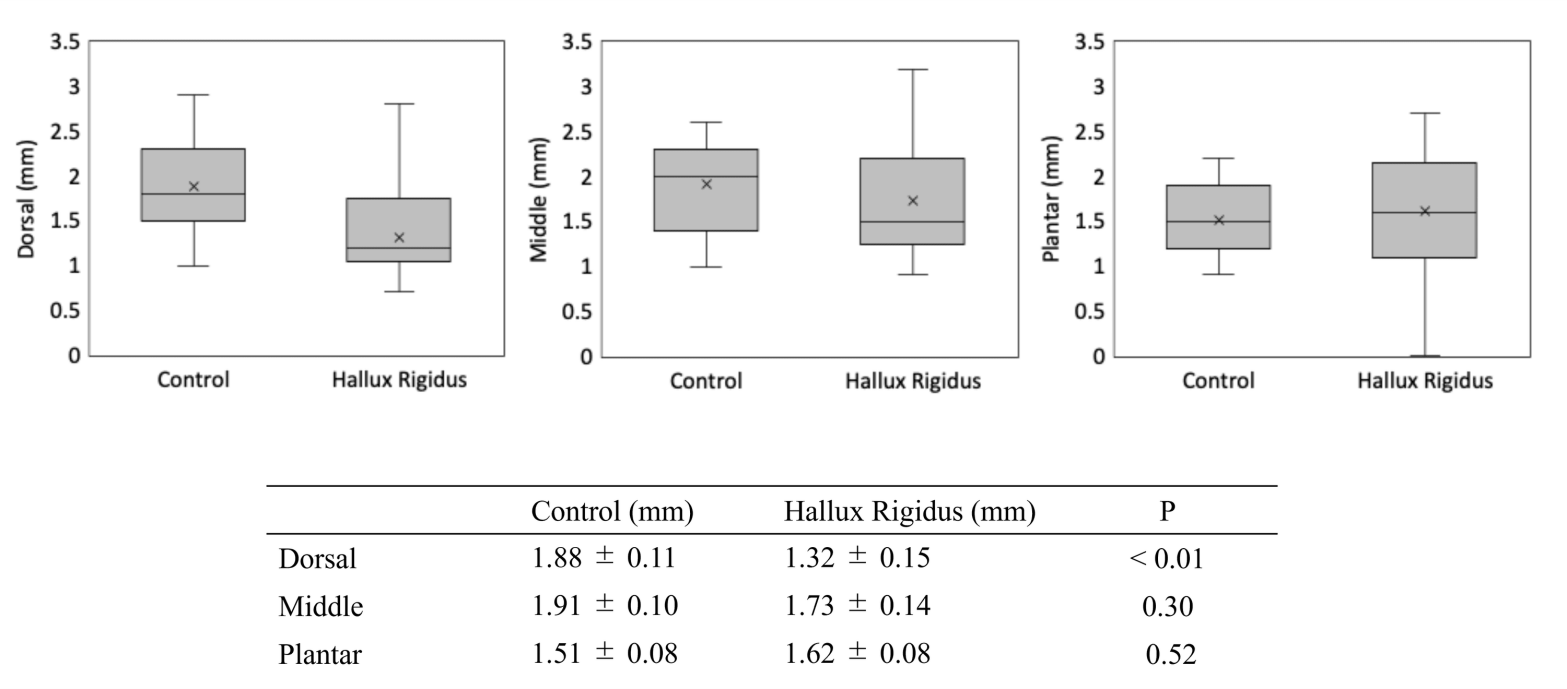
MTP joint space width in the control and hallux rigidus groups. The dorsal joint space was significantly narrower in the hallux rigidus group (P < 0.05).

At the dorsal aspect of the joint, the mean joint space width was 1.32 ± 0.15 mm in the HR group and 1.88 ± 0.11 mm in the control group, showing a significant difference between the groups (P < 0.01). In contrast, the mean joint space width in the central region was 1.73 ± 0.14 mm in the HR group and 1.91 ± 0.10 mm in the control group, and in the plantar region was 1.62 ± 0.08 mm in the HR group and 1.51 ± 0.08 mm in the control group; no significant differences were observed at these locations (Figure 2).

**Figure 3:**
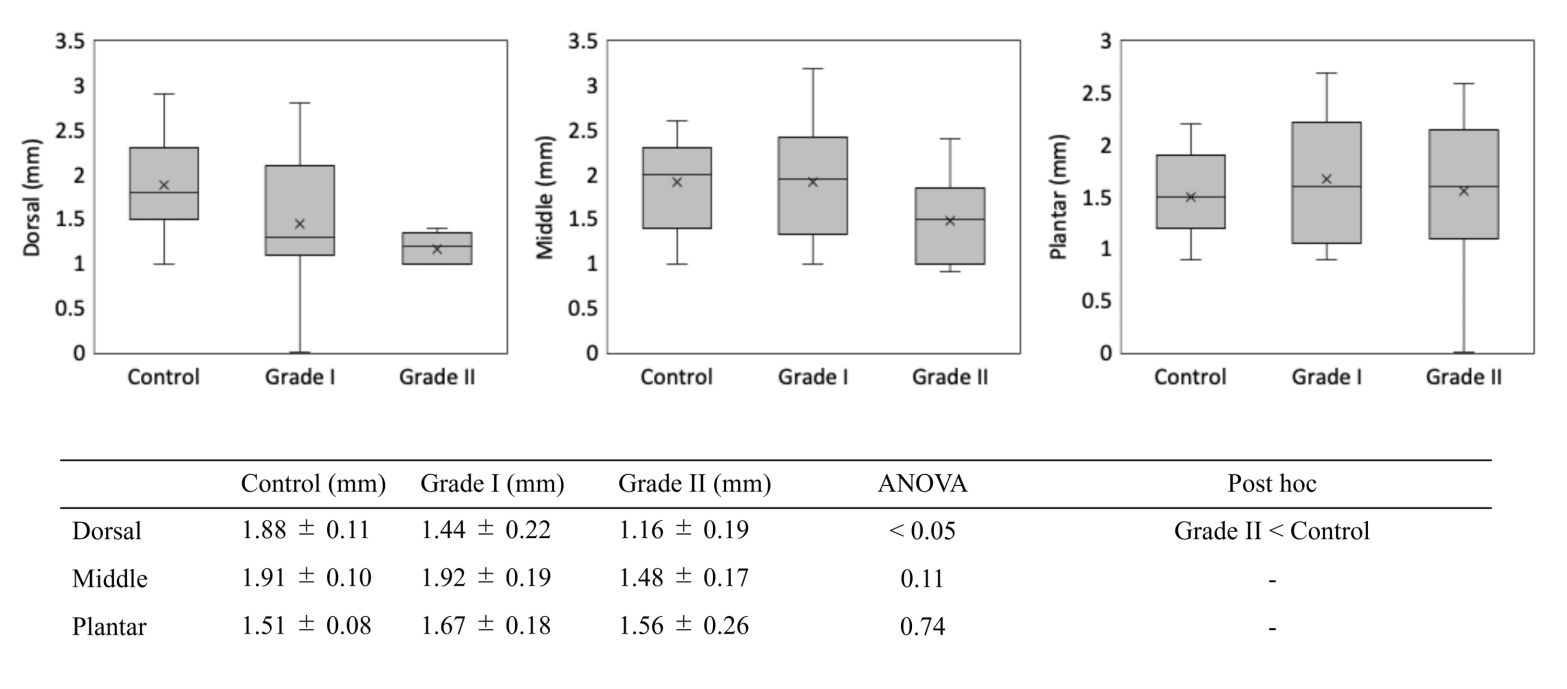
MTP joint space width in the control, Hattrup Grade I, and Hattrup Grade II groups. In the Grade II group, the joint space was significantly narrowed and the affected area extended in the plantar direction. ANOVA: analysis of variance

When compared by disease stage, the dorsal joint space width was 1.44 ± 0.22 mm in Grade I and 1.16 ± 0.19 mm in Grade II. A significant difference was observed between the Grade II and control groups (P < 0.01). On the other hand, no significant differences were found between the control group and any stage of disease in the central or plantar regions (Figure 3).

Deviation of the center of rotation

**Figure 4:**
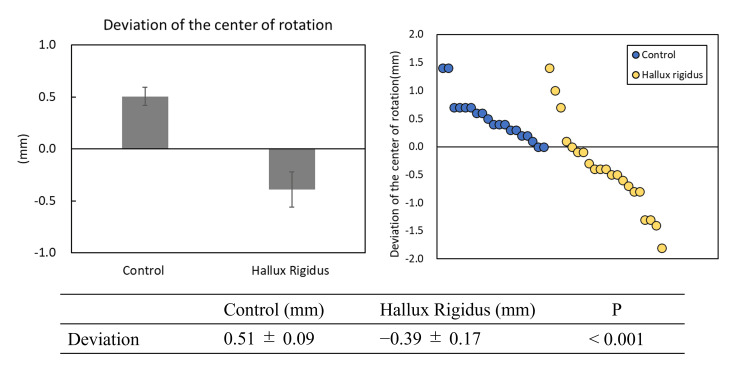
Deviation of the center of rotation. The control group (blue) showed a distribution in the positive direction, while the hallux rigidus group (yellow) showed a deviation primarily in the negative direction. On the x-axis, individual measurements for each group are plotted in descending order of value; the axis does not represent a continuous variable.

The mean deviation of the center of rotation was 0.51 ± 0.09 mm in the control group and -0.39 ± 0.17 mm in the HR group, showing a significant difference (P < 0.01) (Figure 4).

**Figure 5:**
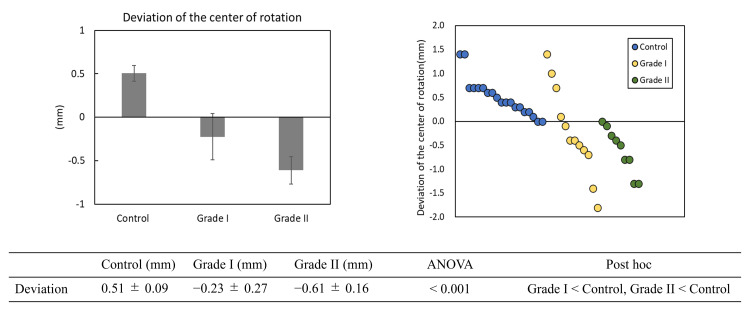
Deviation of the center of rotation deviation according to disease stage. The proximal phalanx was dorsal in all participants in the control group. The Grade I group showed greater variability with a tendency toward plantar deviation, while the Grade II group consistently showed plantar positioning. As in Figure 4, the x-axis plots individual measurements for each group in descending order of value; it does not represent a continuous variable. ANOVA: analysis of variance

By disease stage within the HR group, the mean deviation was -0.23 ± 0.27 mm in the Grade I group and -0.61 ± 0.16 mm in the Grade II group. The Grade I and Grade II groups showed significant differences compared to the control group (P < 0.01). However, there was no significant difference between the Grade I and Grade II groups (Figure 5).

## Discussion

Pain with dorsiflexion of the MTP joint is a characteristic feature of hallux rigidus, particularly in the early to mid stages. As the disease progresses, the MTP joint space narrows, osteophytes form, and range of motion becomes progressively limited, eventually leading to the “rigid” state that defines the condition [7]. In advanced stages, autoankylosis of the joint may occur, which can result in reduced or absent pain; however, this is not observed in all cases [8].

Morphologic features of the foot associated with hallux rigidus include a square or flat shape of the metatarsal articular surface, a structurally or functionally longer first ray compared to the second, and hypermobility or adduction of the first metatarsal. The first ray may appear longer either due to an anatomically longer first metatarsal or because of the rigid combination of the first metatarsal and proximal phalanx in advanced stages. Additional associated conditions include hallux valgus, hallux interphalangeus, and flatfoot.

Reported extrinsic factors include repetitive microtrauma, abnormal gait patterns, footwear, and lifestyle factors [9]. While these may contribute to the development of hallux rigidus, it is also possible that some of these changes arise secondarily as adaptations to early joint dysfunction. Given this complexity, hallux rigidus is considered to have a multifactorial etiology, although many aspects of its pathology remain unclear.

In this study, we focused on joint space narrowing and center of rotation deviation [10] in patients with hallux rigidus and performed a comparative investigation of these changes using weight-bearing CT. Although the first MTP joint may exhibit multiple centers of rotation depending on the degree of dorsiflexion [5], our analysis was based on static weight-bearing CT images obtained with the hallux in a minimally dorsiflexed position. This approach enabled a standardized and reproducible evaluation but represents only one phase of joint motion and does not capture dynamic changes throughout the full range of dorsiflexion.

Joint space narrowing

**Figure 6:**
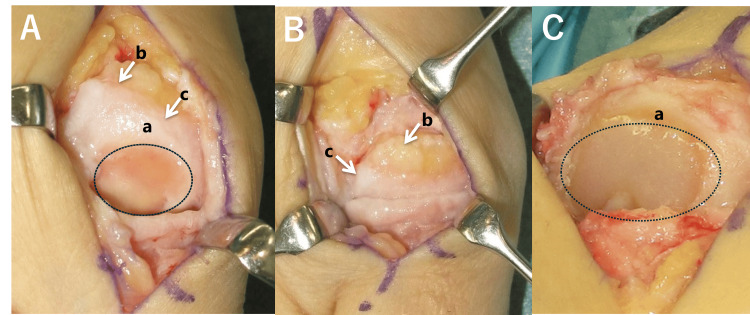
Intraoperative findings. (A) Hattrup grade II with MTP joint plantarflexion; (B) Hattrup grade II in dorsiflexion: A cartilage defect (a) was observed at the metatarsal head with dorsal osteophyte formation (b) and degenerated cartilage (chondrophyte) (c) on the dorsal aspect; (C) Hattrup grade III: A wider cartilage defect (a) was present, extending into the plantar region.

Intraoperative findings in hallux rigidus reveal intra-articular dorsal osteophytes of the first metatarsal head with degenerated cartilage (chondrophytes) at the distal ends of the osteophytes. During dorsiflexion of the MTP joint, these chondrophytes impinge on the base of the proximal phalanx. In addition, cartilage defects are observed at the distal end of the chondrophytes, while healthy cartilage remains intact on the plantar side of the defect. Osteophyte formation and cartilage loss are also seen on the dorsal articular surface of the proximal phalanx (Figure 6). MRI findings show bone marrow edema in the dorsal aspects of the metatarsal head and proximal phalanx.

These observations suggest that in hallux rigidus, there is severe impingement between the base of the proximal phalanx and the dorsal aspect of the metatarsal head [11]. As a result, osteophytes form on the proximal side of the impingement area. In Grade II cases, the cartilage defect is localized to the dorsal side of the metatarsal head, while in Grade III cases it extends to the tip of the head.

Although Grade I hallux rigidus is typically characterized by symptoms without radiographic changes, this study observed a trend toward dorsal joint space narrowing even in Grade I cases, although it was not statistically significant. As the disease progresses, this tendency becomes significant, with joint space narrowing progressing in Grade II and spreading further in the plantar direction. These findings are consistent with the above-described intraoperative observations.

Deviation of the center of rotation

A characteristic feature of hallux rigidus is the dorsal protrusion of the first metatarsal head, commonly referred to as MPE. Since it was first reported by Lambrinudi [12], this phenomenon has been a long-standing topic of discussion regarding the etiology of hallux rigidus. Coughlin and Shurnas considered MPE to be a secondary change as it tends to worsen with disease progression [9]. However, in clinical practice, dorsal protrusion of the metatarsal head is observed even in mild cases without significant osteophyte formation [13]. Coughlin and Shurnas defined MPE as “dorsal displacement of the first metatarsal relative to the second metatarsal” [9], but in this study, MPE was evaluated from the perspective of “dorsal protrusion of the metatarsal head” based on the deviation of the center of rotation.

Under weight-bearing conditions, the proximal phalanx was positioned superiorly in all participants in the control group (mean: 0.51 mm), whereas it was inferiorly positioned in the hallux rigidus group (mean: -0.39 mm), showing a significant difference between the groups. This tendency became more pronounced with disease progression; although the variation was large in the Grade I group, a general downward trend was observed, and in the Grade II group, the center of rotation was located on the plantar side in all cases. The variation in the Grade I group may be related to the influence of uneven weight distribution, as suggested in recent reports, which affects the positional relationship between the first and second metatarsals [14]. When the first metatarsal head is relatively elevated, the proximal phalanx must plantarflex and shift its center of rotation downward to make contact with the ground at the tip of the toe. Such compensation may contribute to fixed MPE, a pattern often observed in advanced hallux rigidus.

In addition, the plantar joint space width in the hallux rigidus group did not narrow as much as in the dorsal or central regions, and in fact tended to be wider than in the control group. This finding suggests that the plantar cartilage may be preserved and that the shift in the center of rotation caused by MPE may result in increased distance on the plantar side.

Recent studies using weight-bearing CT have reported that MPE is already present in the early stages of hallux rigidus and increases with disease severity [15,16]. In a prospective analysis of patients with symptomatic hallux rigidus, Cheung et al. found reduced first metatarsal declination in more advanced radiographic grades [15], while Lee et al. demonstrated significantly greater MPE in the hallux rigidus group and identified a diagnostic threshold of 4.19 mm [16]. Previous analyses of weight-bearing CT images also support these observations [1,2]. Taken together with the results of the present study, these findings suggest that MPE is not merely a secondary change but may be involved in the initial pathogenesis of the disease, warranting a reevaluation of the conventional understanding of hallux rigidus.

Pathophysiology of hallux rigidus

We hypothesize that the breakdown of the windlass mechanism and the presence of MPE are central to the pathophysiology of hallux rigidus. In previous CT imaging studies [1,2], dysfunction of the windlass mechanism was observed even in mild cases of the condition. The windlass mechanism is the process by which the plantar fascia tightens during dorsiflexion of the MTP joint, resulting in elevation of the arch of the foot. When the hallux MTP joint was dorsiflexed under relaxed conditions with the full weight of the foot, medial longitudinal arch elevation was inhibited relative to the normal MTP joint [1,2]. This was considered attributable to a lowered medial longitudinal arch during static weight-bearing.

Additional radiographic features of the foot, such as a square or flat shape of the metatarsal articular surface, a longer first metatarsal compared with the second one, hypermobility or adduction of the first metatarsal, and associated conditions such as hallux valgus, hallux interphalangeus, and flatfoot, suggest that the first metatarsal itself is oriented more horizontally, indicating flattening of the medial longitudinal arch [17].

In the presence of medial longitudinal arch flattening and hypermobility of the first TMT joint, the windlass mechanism becomes dysfunctional and MPE develops [9,18]. When the proximal phalanx dorsiflexes while being pulled downward due to MPE, joint congruity is disrupted, causing concentrated pressure on a limited area of the metatarsal head. This leads to osteophyte formation on the dorsal aspect of the collision site. As the MPE worsens, the collision site between the proximal phalanx and the metatarsal head shifts in a plantar direction, and cartilage loss progresses in that direction.

The hypothesized pathophysiological process proposed by Kihara et al. is believed to largely explain the findings observed in this study and during surgery [2].

Limitations

This study has several limitations. First, it focused primarily on relatively early-stage hallux rigidus cases classified as Grade I or II according to the Hattrup and Johnson classification to investigate factors involved in the onset of the disease. Therefore, the results may not be generalizable to severe cases in which secondary changes are more advanced. Second, all observations were made under static conditions without evaluation under dynamic conditions. Recent studies have reported significant involvement of the intrinsic foot muscles in the windlass mechanism, indicating the need to consider dynamic changes. Third, although intra-observer variability was not formally quantified, anatomical landmarks were identified using multi-planar CT slices, and some measurements were rechecked for consistency in consultation with other orthopedic surgeons when necessary. Finally, this study focused only on the hallux and did not investigate the influence of other toes or the forefoot as a whole on the windlass mechanism.

Clinical implications

Considering these mechanisms, cheilectomy, which is currently widely performed, may not fundamentally resolve the underlying pathology. The reoperation rate is generally reported to be 5-10%, and outcomes are particularly poor in severe cases (classified as grade III in the Hattrup and Johnson system) [19]. Therefore, future treatment interventions should consider ways to appropriately regulate the tension of the plantar fascia and intrinsic foot muscles and to ensure the stability of the medial longitudinal arch.

## Conclusions

This study demonstrated that in hallux rigidus, cartilage loss at the dorsal aspect of the joint and MPE, interpreted as a deviation of the center of rotation under load, are present in the early stages of the disease. As these changes progress, they may further disrupt joint congruity and promote osteophyte formation and arthritic changes.
